# Hospital electronic prescribing system implementation impact on discharge information communication and prescribing errors: a before and after study

**DOI:** 10.1007/s00228-017-2274-7

**Published:** 2017-06-22

**Authors:** Pamela Ruth Mills, Anita Elaine Weidmann, Derek Stewart

**Affiliations:** 1 University Hospital Crosshouse, Pharmacy Department, Kilmarnock, Ayrshire, KA2 OBE Scotland UK; 2 School of Pharmacy and Life Sciences, Robert Gordon University, Aberdeen, AB10 7GJ Scotland UK

**Keywords:** HEPMA, Prescribing error, Sociotechnical error

## Abstract

**Purpose:**

The study aimed to test the hypothesis that hospital electronic prescribing and medicine administration system (HEPMA) implementation impacted patient discharge letter quality, nature and frequency of prescribing errors.

**Method:**

A quasi experimental before and after retrospective case note review was conducted in one United Kingdom district general hospital. The total sample size was 318 (random samples of 159 before and after implementation), calculated to achieve a 10% error reduction with a power of 80% and *p* < 0.05. Adult patients discharged after ≥24-h inpatient stay were assessed for discharge information documentation quality using a modified validated discharge document template. Prescribing errors were classified as medicine omissions, commissions, incorrect dose/frequency/duration, drug interactions, therapeutic duplications or missing/inaccurate allergy information. Post-implementation assessments were undertaken 4 months following HEPMA implementation. Error severity was determined by a multidisciplinary panel consensus using the Medications at Transitions and Clinical Handoffs (MATCH) study validated scoring system.

**Results:**

There were no statistically significant differences in patient demographics between the pre- and post-implementation groups. Discharge information documentation quality improved; allergy documentation increased from 11 to 159/159 (*p* < 0.0001). The number of patients with prescribing errors reduced significantly from 158 to 37/159 (*p* < 0.001). Prescribing error category incidence identified in pre-implementation patients was reduced (e.g. omission incidence from 66 to 18/159 (*p* < 0.001)), although a new error type (sociotechnical [errors caused by the system]) was identified post-implementation (*n* = 8 patients). Post-implementation prescribing errors severity rating identified 8/37 as likely to cause potential patient harm.

**Conclusion:**

HEPMA implementation was associated with improved discharge documentation quality, statistically significant prescribing error reduction and prescribing error type alteration. There remains a need to be alert for potential prescribing errors.

## Introduction

Hospital electronic prescribing and medicines administration (HEPMA) systems were defined by the Department for Health in England in 2007 as ‘the utilisation of electronic systems to facilitate and enhance the communication of a prescription or medicine order, aiding the choice, administration and supply of a medicine through knowledge and decision support and providing a robust audit trail for the entire medicines use process’ [1]. UK government strategy recommends HEPMA system implementation, with National Health Service (NHS) England and NHS Scotland policies committed to HEPMA as a future e-health model in all secondary healthcare settings [2, 3].

Reported advantages of electronic prescribing systems include increased patient safety by prescribing error reduction. The UK Health Foundation stated that implementation of an electronic prescribing system incorporating decision support functionality could realise a 50% prescribing error reduction [4]. These systems also have the capacity to create prescriptions at the point of patient discharge from secondary care [5]. Electronic systems are, not without their limitations, having been shown to lead to ‘sociotechnical errors’, defined as those ‘occurring at the point where the system and the professional intersected and would not have occurred in the absence of the system’ [6].

In the UK, implementation of electronic prescribing systems is variable. A survey of English hospitals in 2011 reported that while 48% of hospitals had implemented standalone electronic discharge systems, only 13% had implemented integrated inpatient and discharge electronic prescribing [7]. Reduction in inpatient prescribing errors has been demonstrated with electronic prescribing systems [8, 9]. However, there has been limited study of prescribing error alteration in discharge prescriptions. A narrative literature review of discharge information communication and medicines discharge prescribing errors identified four studies comparing electronic solutions to traditional handwritten systems and five of electronic solutions with no comparison. These studies demonstrated inconsistent results with improved, unchanged or reduced medicine information accuracy [10]. Notably, none of the study sites had implemented the UK HEPMA systems.

Healthcare Improvement Scotland state that HEPMA systems need to be at least as safe as the traditional paper systems they replace [11]. Guidance on hospital medicines discharge documentation requirements are provided by two UK bodies, the Royal Pharmaceutical Society (RPS) and the Scottish Intercollegiate Guideline Network (SIGN) [12, 13]. The RPS guidance proposes core content for medicines information communication on hospital discharge, including a mandatory requirement for medicines change information to be recorded [12]. SIGN guidance defines the ideal content of hospital discharge documentation including a standard template with 29 required sections [13]. In 2014, NHS England identified specific issues concerning discharge medicines information communication which resulted in widespread dissemination of a patient safety alert to highlight the importance of essential information communication on discharge [14]. Medicine-related events are the most common adverse event occurring following hospital discharge, with evidence of perpetuation of discharge prescribing errors associated with a moderate potential for harm [15]. Failure to recognise these adverse events is a contributory factor for hospital readmission [16].

Currently, there is a lack of published evidence relating to HEPMA impact on discharge medicines information communication and prescribing error prevalence.

The aim of this study was to test the hypothesis that HEPMA implementation impacted discharge letter quality, nature and frequency of prescribing errors.

## Methods

### Design

A quasi experimental, before and after retrospective case note review design was adopted.

### Setting

The study was undertaken in a 560 bedded district general hospital (DGH) in the UK. The selected HEPMA system was a commercially available standalone system; HEPMA implementation commenced in October 2013 and was completed in September 2014. Prior to HEPMA implementation, manual handwritten immediate discharge letters (IDLs) were prepared by either a medical prescriber or an advanced nurse practitioner transcribing diagnostic information from handwritten case notes and inpatient chart(s). Post-HEPMA implementation, both the inpatient prescription chart and the IDL were prepared electronically while the case notes remained handwritten. The IDL is prepopulated with information such as demographics and allergies. Discharge medicines can be selected from those prescribed as an inpatient and those to be commenced at the point of discharge added. Diagnostic and other clinical information is added by the clinician completing the IDL.

### Sampling

The sample size calculation was based on anticipated baseline error rate of 17.5% [10]. The aim was to achieve a 10% error reduction with a power of 80% and *p* < 0.05. The total sample size was 318, comprising random samples of 159 before and after implementation.

Patient inclusion criteria were as follows:

- Discharged between April to June 2013 (pre) and February to April 2015 (post)
- ≥16 years
- Discharged from hospital after an inpatient stay of at least 24 h

Patient exclusion criteria were as follows:

- Mental health, maternity and paediatric wards (HEPMA not implemented or implemented prior to the study)
- No medicines prescribed at discharge
- Inter-hospital transfer

### Pre-implementation data collection

Case notes were reviewed retrospectively by the principal investigator, an experienced clinical pharmacist, with a focus on information content and presence of prescribing errors on the handwritten IDL by scrutinising clinical notes, inpatient prescription charts and IDLs. Data were extracted onto a data collection tool, which was a modified version of the SIGN discharge document template [13]. Variables documented were age, gender, general practitioner details, hospital consultant details, discharge ward, discharge specialty, length of stay, discharge day of week, primary diagnosis, secondary diagnosis, significant operations/procedures, allergies, number of medicines prescribed on IDL, and designation of clinician completing and IDL. An independent clinical pharmacist confirmed the reliability of data extraction in a random sample of 10% of patients.

Data of primary diagnosis, secondary diagnosis and significant operations/procedures were not assessed for accuracy, merely recorded as present or absent in accordance with the approach of Callen et al. [17].

Medicines prescribed on discharge were compared to the inpatient list to identify any discrepancies.

The definition of prescribing error of Dean et al. was adopted, ‘a prescribing error occurs, when as a result of a prescribing decision or prescription writing process, there is an unintentional reduction in the probability of treatment being timely and effective or increase in the risk of harm’ [18]. Prescribing error types were classified as medicine omission, medicine commission, incorrect dose, incorrect frequency, incorrect duration, drug interaction, therapeutic duplication, missing allergy and inaccurate allergy, as described in Table 1. Reliability of error categorisation in a random sample of 10% was confirmed by an independent clinical pharmacist.

**Table 1:** Error type classification

Error type	Description	Exclusion
Omission	Medicine omitted from IDL currently prescribed on inpatient chart. Medicine administered preceding/discharge day. For example documentation of ‘no changes to routine medicines’.	Medicine not usually required on discharge, for example antiemetic injection.
Commission	Medicine prescribed on IDL not on pre-admission list. Medicine not administered preceding/discharge day, e.g. cyclizine (antiemetic) prescribed as a precaution but never administered.	Explanatory note documented for medicine requirement.
Incorrect dose	Discrepancy between dose on inpatient chart and IDL or no dose documented, e.g. carvedilol noted as 19 mg instead of 18.75 mg	Explanatory note documented regarding dose change.
Incorrect frequency	Discrepancy between frequency documented on inpatient chart and IDL or none documented. For example, as required medicines prescribed without specified time interval.	Explanatory note documented regarding frequency change.
Incorrect duration	Discrepancy between duration documented on inpatient chart and IDL or no documented duration provided.	Explanatory note documented regarding duration change.
Drug interaction	A drug interaction recorded as a serious interaction in current edition of British National Formulary.	Appropriate to co-prescribe with suitable monitoring.
Therapeutic duplication	More than one medicine prescribed from same therapeutic group. Co-codamol and tramadol co-prescribed.	Protocol exists to evidence prescribing action.
Missing allergy	Allergy documented on inpatient chart and/or patients’ case notes but not on IDL. Nil known drug allergy (NKDA) missing from IDL.	Explanatory note documented regarding allergy information.
Inaccurate allergy	Discrepancy between allergy documented on inpatient chart and/or patients’ case notes and IDL.	Explanatory note documented in case note regarding allergy information change.
Sociotechnical (post-HEPMA)	Error caused by HEPMA system, e.g. prednisolone soluble tablets instead of plain tablets.	Error unlikely to be caused by HEPMA.

### Post-implementation data collection

A similar method was adopted for the post-implementation phase. Prescribing errors were identified and categorised as before, with the addition of sociotechnical errors in the post-implementation group. Examples of sociotechnical errors include incorrect selection of medicine formulation [6, 19]. Following data extraction, one medical consultant and one clinical pharmacist scored prescribing error severity. The panel was provided with information about each prescribing error as per previous studies [20]. The panel were also provided with a copy of the severity scoring guidance which consisted of a validated scoring system developed by Gleason et al. [21]. The severity ratings were as follows:

1. No potential harm
2. Monitoring or intervention potentially required to preclude harm
3. Potential harm

The panel met and discussed each error in turn, assigning a severity score. If consensus was not achieved, the error was referred to an independent medical consultant for further assessment.

### Data analysis

Data were input to Statistical Packages for Social Sciences (SPSS) version 21.0, and analysed using descriptive statistics, normality tests and inferential statistics [22]. The Shapiro-Wilk normality test was applied to determine data distribution; Mann Whitney *U* test was used for non-parametric variables including patient length of stay and discharge specialty. Categorical variables were analysed using chi-square test for data with values greater than zero and Fisher exact test for data including a count of zero. The accepted level for significance was *p* < 0.05.

### Governance

The study was approved by the ethical review panel of a UK university; NHS ethics was not required as it was considered a ‘service evaluation’. Caldicott guardian approval was obtained to access patient confidential information [23].

## Results

Data were found to follow a non-parametric distribution. There were statistically significant differences between groups as depicted in Table 2 (*p* > 0.05). The median age was 60 years pre-implementation and 59 post-implementation, with gender consistent at 57% female. The most common length of stay was 2 days for both groups and discharge specialty was consistent with 47% medical specialties; more medicines were prescribed post-implementation (*p* = 0.023). There was a statistically significant improvement in some aspects of documentation (patient’s GP details, allergy information, grades of staff); no impact on others (hospital consultant, relevant secondary diagnosis, signature and full name printed); while certain sections were associated with reduced information documentation (diagnosis and procedures/operations) (Table 3).

**Table 2:** Comparison of pre- and post-implementation results

Variable	Pre-implementation (*n* = 159)	Post-implementation (*n* = 159)	Significance *p* value
Age range (years)	18–102 Median 60	17–93 Median 59	0.416
Gender	57% female	57% female	
Length of stay (days)	1–25 Mode 2	1–33 Mode 2	0.232
Discharge specialty	Medical 47% Surgical 33%	Medical 47% Surgical 30%	0.688
Range of number of discharge medicines	0–25 Median 5.0	0–18 Median 6.0	0.023
Total number of IDL prescribed medicines	872	1018	0.023

**Table 3:** Comparison of documented information on IDL

Comparison of number and percentage of patients with required information	Pre-HEPMA *n* = 159 N (%)	Post-HEPMA *n* = 159 N (%)	Chi-square	*p* value
Patient’s GP details	89 (56.0)	157 (98.7)	83.019	<0.001
Hospital consultant	154 (96.9)	159 (100)	Fisher exact*	0.0605
Diagnosis	153 (96.2)	116 (73.0)	33.028	<0.001
Relevant secondary diagnosis	48 (30.2)	49 (30.8)	0.015	0.902
Procedures/operations	99 (62.3)	62 (39.0)	17.223	<0.001
Allergy information	11 (6.9)	159 (100)	Fisher exact	<0.0001
Signature	159 (100)	159 (100)	Fisher exact	1.0
Full name printed	157 (98.7)	159 (100)	Fisher exact	0.4984
Grade of staff	64 (40.2)	159 (100)	Fisher exact	<0.0001

Table 4 gives the comparison of prescribing errors pre- and post-HEPMA implementation. There was a statistically significant reduction in the number of patients with a prescribing error post-HEPMA implementation, a reduction from 158 (99.4%) to 37 (23.3%) patients (*p* < 0.001.). The prevalence of all error types was reduced, being statistically significant for omitted medicines, medicine commission, incorrect doses, incorrect frequencies, incorrect durations and missing allergies (Table 4). Sociotechnical errors were attributed to prescriber selection failure for formulations and administration route for eye drops. Multiple error types were detected in 41.5% (*n* = 66) pre-implementation patients reduced to 2% (*n* = 3) post-implementation. Multiple instances of the same error (for example, omitted medicines) occurred in 56% (*n* = 89) pre-implementation patients reduced to 7% (*n* = 11) post-implementation.

**Table 4:** Comparison of prescribing errors

Comparison of number and percentage of patients with prescribing errors	Pre-HEPMA *n* = 159 N (%)	Post-HEPMA *n* = 159 N (%)	Chi- square	*p* value
Patients with errors including omitted allergy information	158 (99.4)	37 (23.3)	194.115	<0.001
Patients with errors excluding NKDA	134 (84.3)	37 (23.3)	119.03	<0.001
Omitted medicines	66 (41.5)	18 (11.3)	37.275	<0.001
Medicine commissions	10 (6.3)	1 (0.6)	7.627	0.006
Incorrect doses	14 (8.8)	1 (0.6)	11.824	<0.001
Incorrect frequencies	30 (18.9)	2 (1.3)	27.241	<0.001
Incorrect durations	43 (27.0)	3 (1.9)	40.665	<0.001
Drug interactions	7 (4.4)	1 (0.6)	4.616	0.032
Therapeutic duplications	5 (3.1)	4 (2.5)	0.114	0.736
Missing allergies	154 (96.9)	2 (1.3)	290.72	<0.001
Incorrect allergies	2 (1.3)	0	Fisher exact	0.498
Sociotechnical error	0	8 (5.0)	Fisher exact	0.007

### Error severity scoring

Errors were detected in 23% of post-implementation patients (*n* = 37). Severity scoring results gave category 1 errors (causing no potential harm) in 8 (22%) of patients with errors, category 2 errors (monitoring or intervention potentially required to preclude harm) in 19 (53%) and category 3 errors (likely to cause potential patient harm) in only 8 (22%). Table 5 provides example error descriptions with associated score. Severity score 1 errors included incorrect formulation selected and 5 days of cyclizine supplied on discharge when none had been administered during the inpatient stay.

**Table 5:** Post-HEPMA error severity scoring examples

Error description	Error type	Severity score
Co-prescribed fluoxetine and amitriptyline (only taking amitriptyline prior to admission)	Therapeutic duplication	2
No medicines added to IDL but patient had 18 medicines prescribed and administered as inpatient which should be continued on discharge.	Omission	3
Esomeprazole 40 mg once daily prescribed as inpatient but omitted from IDL.	Omission	2
Wrong formulation of phenoxymethylpenicillin selected; syrup instead of tablets.	Sociotechnical	1
Lantus® and Humulin S® on IDL with no frequency documented. Marked as charted but the insulin chart would not be sent to the patient’s GP.	Incorrect frequency	1
Simvastatin withheld during inpatient stay as co-prescribed clarithromycin. Information documented on IDL to restart simvastatin once antibiotics completed. Simvastatin and clarithromycin both prescribed on IDL and both dispensed.	Drug interaction	2
5-day supply of cyclizine requested on IDL but not administered during inpatient stay.	Commission	1
Clomipramine prescribed in morning but should be at night as per admission medicine reconciliation. (HEPMA defaults to 10 p.m. time).	Incorrect frequency	2
Amiodarone 200 mg tablets selected for 100 mg dose (100 mg tablets available).	Sociotechnical	2
Commenced on zopiclone for night sedation but developed a skin rash so stopped. Information not documented on IDL nor allergy status updated.	Omitted allergy	3
Palliative care recommended codeine and sevredol for pain as tramadol no longer effective but all three on IDL plus dihydrocodeine.	Therapeutic duplication	3
Meloxicam, azathioprine and sulfasalazine should be restarted at normal doses 1-week post-discharge but none prescribed on IDL and not mentioned on IDL.	Omission	3
Tranexamic acid should be continued until clinic appointment but marked as 28-day supply with GP to continue.	Incorrect duration	2
Omeprazole prescribed as gastrointestinal cover while on diclofenac but information not communicated to GP so potential could be continued.	Incorrect duration	3
Allergy information recorded as other (see medical notes). There was an inpatient note documented as sodium benzoate causes mouth ulcers but note not added to the IDL.	Missing allergy	2

Assessors agreed for all severity scoring.

The null hypothesis was rejected; HEPMA implementation positively impacted discharge letter quality, with a reduction in prescribing error frequency and severity of errors.

1=no potential harm, 2=monitoring or intervention potentially required to preclude harm, 3=potential harm

## Discussion

### Key findings

The key study findings relate to discharge letter quality and the nature and type of prescribing errors. HEPMA implementation resulted in an improvement in information content and accuracy. An improvement for almost all assessed SIGN guideline criteria was a consequence of HEPMA implementation [13]. HEPMA implementation significantly reduced the number of patients with prescribing errors (*p* < 0.001). Allergy information documentation improved with missing allergy information practically eliminated (*p* < 0.0001). Medicine omission was the most frequent error type post-implementation, although the incidence was significantly reduced compared to pre-implementation levels (*p* < 0.001). Errors related to the system (sociotechnical errors) were detected in 5% post-implementation patients (*n* = 8). HEPMA prescribing errors were categorised as potentially associated with harm in 5% patients (*n* = 8).

### Strengths and weaknesses

The study strengths include application of a consistent approach by use of an adapted validated tool and appropriate study design to minimise bias wherever possible. Biases minimised were measurement bias by use of a validated tool and non-response and sampling biases by use of a random patient sample and by systematic application of the sample. There are, however, several limitations hence the study findings should be interpreted with caution. While a randomised control trial would be the ideal study design, this was not possible due to the nature of HEPMA implementation within the study setting. Other limitations were the before and after design which resulted in different patients in the two phases. Severity scoring assessment is noted to be subjective and the panel was limited to the perspectives of one medical consultant and one senior pharmacist. There may have been other confounders between the two study periods impacting the findings. These included changes in medical staff and other changes in operational processes. Furthermore, there was no consideration of actual patient harm as a result of prescribing errors.

### Interpretation

This study demonstrated improvements to discharge information content and accuracy while previous studies had reported inconsistent results when moving to electronic systems, although disparate methods were employed in the different studies [17, 24–26]. The documentation of diagnosis in the designated section of the IDL was reduced although frequently, this information was incorporated into the clinical progress section. A similar finding was reported by Callen et al. when they compared electronically produced letters to handwritten ones [24]. Prescribing error frequency was reduced as a consequence of HEPMA implementation. HEPMA confers automatic import of information from the inpatient chart to the IDL which is consistent with a recommendation by Kriplalani et al. that ‘hospitals should use information technology to extract information into discharge summaries to ensure accuracy (e.g. medication names and doses) and to facilitate rapid completion of summaries’ [27]. HEPMA implementation eradicates medicine transcription for IDLs which was predicted to reduce prescribing errors and increase the total number of medicines prescribed on IDLs [17, 24]. Grimes et al. in an observational study reported medicine discrepancies in 66% patients at hospital discharge [28]. HEPMA implementation reduced prescribing errors from 84 to 23% patients (excluding omitted allergy information). The most frequent post-implementation prescribing error type was omitted medicines, as demonstrated in retrospective studies focusing on electronic discharge systems [17, 24, 28–30]. Sociotechnical errors accounted for 10 (13%) of post-implementation errors and therefore the HEPMA system prevented more errors than it created. This is consistent with inpatient electronic prescribing error occurrence detected by incident report review [6]. Errors occurring as a consequence of making changes to inpatient charts after preparation of IDLs have been reported when transcribing information from paper inpatient to electronic discharge letters [24]. A similar error was detected post-HEPMA, despite a system alert to indicate that the IDL also required to be changed. Thus, HEPMA implementation has not completely eliminated prescribing errors. The majority of detected prescribing errors were classified as execution errors in Reason’s model (slips or lapses) which generally occur due to human fallibility [20]. Hence, evidence of planning failures remained where practitioners considered their erroneous actions to be correct.

Comparison with published studies indicates that error severity is lower with HEPMA compared to traditional handwritten processes. Published error severity varied and a range of severity scoring assessments were utilised. Grimes et al. reported error severity rates in handwritten IDLs as 47% no harm or minor potential harm, with 53% as moderate potential patient harm [28]. McMillan et al. assessed 88% of errors as minor or potentially troublesome for handwritten discharge letters [31]. Abdel-Qader et al. categorised discharge electronic prescribing errors as serious 2.9%, significant 76.3% and minor 20.8% [30].

## Conclusion

This study has provided evidence that HEPMA implementation in a UK DGH hospital was associated with a statistically significant reduction in discharge prescribing errors and severity of prescribing errors with a concurrent improvement in discharge information content. The electronic system is not a panacea for prescribing errors as system-related errors were detected although with a lower error severity than pre-existing error types.
